# Mindreading beliefs in same- and cross-neurotype interactions

**DOI:** 10.1177/13623613231211457

**Published:** 2023-11-18

**Authors:** Elizabeth Sheppard, Sophie Webb, Helen Wilkinson

**Affiliations:** University of Nottingham, UK

**Keywords:** autism, double empathy, mindreading beliefs

## Abstract

A large sample of autistic and non-autistic adults was recruited to investigate whether self-reported beliefs about their own and other people’s mindreading abilities were in line with either mindreading deficit accounts of autism or the double empathy problem (DEP) (which proposes mindreading difficulties are relational in autism). Three hundred and forty-eight (139 autistic) participants completed an online questionnaire which asked about autism identification and diagnostic status and their beliefs about their own mindreading abilities in relation to autistic and non-autistic others, and about autistic and non-autistic others’ abilities to read their (the participant’s) own minds. While autistic participants did report weaker mindreading abilities, this was only true in relation to non-autistic others. Both groups reported better mindreading abilities in same- than other-neurotype interactions, with autistic participants reporting mindreading abilities commensurate with non-autistic participants in relation to autistic targets. The same pattern was found when participants were asked about other people’s mindreading abilities. The findings were more consistent with the DEP than deficit theories, as the target reference group strongly impacted participants’ perceived mindreading abilities. While self-reported beliefs do not necessarily reflect actual mindreading abilities, they may still have important consequences for intergroup relations and social outcomes for autistic people.

**Lay Abstract:**

Autistic people are often characterised as having problems with mindreading, which refers to understanding other people’s thoughts, beliefs and feelings. However, it has recently been suggested that mindreading difficulties may be a two-way issue between autistic and non-autistic people. This would imply that autistic people may not have difficulty reading the minds of other autistic people, whereas non-autistic people may struggle to read autistic people effectively. In this study, we created a survey in which we asked a relatively large sample of autistic and non-autistic people to rate their own and others’ mindreading abilities in relation to autistic and non-autistic others, respectively. Both groups believed that they were better at reading others in their own group than the other group. The autistic respondents reported levels of mindreading skill at least commensurate with the non-autistic respondents when the mind to be read was specified as autistic. Thus, both groups of participants’ responses were consistent with the notion that mindreading abilities are relational. Although self-reports are subjective, such beliefs could have important consequences for well-being and intergroup relations.

Autism is formally diagnosed based on social and communicative difficulties as well as restricted interests/engagement in repetitive behaviours (American Psychiatric Association, 2013). Historically, the social differences associated with autism were accounted for by an ‘impairment’ in the ability to read others’ minds (also known as ‘theory of mind’, ‘mentalizing’ or ‘mindreading’;^1^ Baron-Cohen et al., 1985). A substantial amount of research has provided empirical evidence that autistic people seem to struggle with tasks designed to measure mindreading ability (Boucher, 2012 for a review). However, this theory has increasingly been criticised for failing to take the bi-directional nature of social interaction into account, with research until recently almost exclusively focusing on the abilities of autistic people to interpret the behaviour of non-autistic others while failing to consider the converse issue (Milton et al., 2022).

In contrast, the double empathy problem (DEP; Milton, 2012) proposes that in interactions between autistic and non-autistic individuals, there are mutual failures in mindreading which arise from the very different ways in which the world is experienced by these two populations. A key novel prediction which can be derived from the DEP (compared with mindreading deficit theories) is that non-autistic people will have more difficulty reading the minds of autistic people than of other non-autistic people (Milton, 2012). A second prediction that arguably may be derived from the DEP is that, due to their shared experiences and perspectives, autistic people may be better – perhaps at a level commensurate with non-autistic people – at reading the minds of other autistic people (Crompton et al., 2020a; Milton, 2012).

A small body of research has tested these predictions using various experimental methods, although most studies have only tested part of the model, for example, non-autistic perceivers’ ability to read autistic targets. In line with the DEP, several studies have found that non-autistic people do have difficulty identifying facial expressions and interpreting the behaviour of autistic people (Macdonald et al., 1989; Sheppard et al., 2016 although see also Faso et al., 2015 for counterevidence). However, two studies which examined multiple predictions that can be derived from the DEP by including both autistic and non-autistic perceivers and targets found that autistic people did not show an own-neurotype advantage in mindreading performance (Brewer et al., 2016; Edey et al., 2016). In contrast, a study examining informational transfer between same- and mixed-neurotype chains of participants found that transfer between mixed chains was poorer than between non-autistic-only and autistic-only chains, which did not differ from one another (Crompton et al., 2020a). While this study did not directly measure mindreading, it is in line with the notion that autistic people have a greater understanding of other autistic people than non-autistic people generally have.

One possible reason for the mixed findings is that mindreading accuracy tasks lack generalisability (Davis & Kraus, 1997). Cognitive measures of mindreading usually measure just one aspect of mindreading (e.g. facial expression recognition) at a specific timepoint, often bereft of social context. A recent review highlighted that performance on sociocognitive mindreading tasks often fails to replicate, and individual mindreading tasks fail to converge, raising serious questions about the validity of mindreading deficit theories of autism (Gernsbacher & Yergeau, 2019). Moreover, they have not consistently been found to predict outcomes of real-world social interactions, including between autistic and non-autistic people (Begeer et al., 2010). For example, in a study that examined the impressions formed following a brief get-to-know-you conversation between autistic, non-autistic or mixed dyads, standardised measures of social cognition (such as face perception and emotion recognition) had little impact on favourability of the impressions formed (Morrison et al., 2020).

An alternative way to investigate mindreading experiences is to ask participants about their abilities in mindreading in relation to both same and cross-neurotype others. To our knowledge, no previous research has directly asked autistic or non-autistic participants about their perceived mindreading abilities in relation to different target groups. However, a qualitative study which interviewed autistic adults about their relationships with autistic and neurotypical friends and family reported that autistic adults found other autistic people easier to read, as well as having a better understanding of, and feeling better understood by, other autistic people (Crompton et al., 2020b). While the perceptions of autistic people in this study appeared to be in strong concordance with the DEP, it is not clear whether non-autistic adults have similar perceptions – for instance, whether they believe that they themselves have poor mindreading skills in relation to autistic targets.

The current study aimed to explore the mindreading beliefs of a large sample of autistic and non-autistic adults in relation to same- and cross-neurotype interactions. Participants completed a total of four modified versions of the Mind Reading Belief Scale (MBS; Realo et al., 2003) which was originally developed as a measure of an individual’s perceived ability to infer the mental states, emotions, behaviours, characteristics and intentions of others. This scale was chosen due to its focus on perceived mindreading abilities and good psychometric properties (Realo et al., 2003). The wording of the scale was modified to explicitly state the reference group for participants’ judgements, such that one version asked about participants’ perceived ability to read the minds of *autistic* others and one version asked about participants’ perceived ability to read the minds of *non-autistic* others.

While the MBS only asks about participants’ beliefs about their own mindreading ability, in the current research, two further versions were created which asked participants about how well they believed autistic and non-autistic other people could read the participant’s own mind. These new versions asked about the same interactions but from the reverse perspective and were included due to the possibility of group differences in self-enhancement when estimating one’s own abilities (Schriber et al., 2014), which we assumed would be less likely to affect judgements of others’ abilities. Finally, previous research has shown that contact with autistic people impacts attitudes towards and social impressions formed of autistic people by non-autistic others (de Vries et al., 2020; Gardiner & Iarocci, 2014; Sasson & Morrison, 2019) and there are characteristic features of how autistic people interact with one another (Heasman & Gillespie, 2019; Rifai et al., 2022), participants were also asked about the amount of contact they have had with autistic people.

If participants’ beliefs about their mindreading abilities are in line with autism mindreading deficit theories, then (1) non-autistic people would believe that they are better at reading other minds than autistic people; (2) target group (autistic or non-autistic) would have no effect on mindreading beliefs. On the other hand, if participants’ beliefs are in line with the DEP then (3) non-autistic people would believe that they are better at reading non-autistic minds than autistic minds; (4) autistic people would believe that they are better at reading autistic minds than non-autistic minds. For beliefs regarding other people’s mind reading abilities, mindreading deficit theories would predict that (5) people would believe that non-autistic people would read them better than autistic people. In contrast, from the DEP we derived the prediction that (6) non-autistic people would believe that other non-autistic people would read them better than autistic people; (7) autistic people would believe that other autistic people would read them better than non-autistic people. Finally, we tested the prediction that (8) non-autistic and autistic participants who report having had higher levels of previous contact with autistic people would believe that they could read autistic minds more effectively than those who have had lower levels of previous direct contact.

## Method

The entire procedure was approved by the School of Psychology Ethics Committee at the University of Nottingham (Ethics approval number: S1271). All procedures performed in studies involving human participants were in accordance with the ethical standards of the institutional and/or national research committee and with the 1964 Helsinki Declaration and its later amendments or comparable ethical standards. Informed consent was obtained from all participants.

There was no community involvement in the reported study.

### Participants

Three hundred and forty-eight participants were recruited from various websites and social media platforms including public Facebook pages, Reddit pages and http://surveycircle.com. Participation was entirely voluntary, anonymous, and no financial compensation was provided. Some additional respondents started the questionnaire but did not finish it. Any such participants were considered to have withdrawn and their data was not retained. Of this sample, 139 individuals (36 men, 77 women, 23 other, 3 preferred not to say) aged between 18 and 59 years old (M = 28.36, SD = 10.01) formed the autistic group having either identified as autistic (*N* = 134) and/or having previously received a formal diagnosis of autism (*N* = 92). Forty-four participants said that they identified as autistic but did not have a formal diagnosis and one participant identified as autistic but did not wish to say whether or not they had a formal diagnosis. Two participants reported having a formal diagnosis but did not identify as autistic and three reported having a formal diagnosis but preferred not to say whether they identified as autistic. The non-autistic group consisted of 209 participants (43 men, 162 women, 4 others and 1 preferred not to say) aged between 18 and 62 years old (M = 28.68, SD = 10.34). All participants in the non-autistic group stated that they did not identify as autistic and that they had not received a formal diagnosis. A further five participants did not disclose whether they had ever received a formal diagnosis of autism or whether they identified as autistic and were therefore excluded from the data analysis. An independent samples *t*-test revealed the two groups (autistic and non-autistic) did not differ in mean age, *t*(257) = 0.22, *p* = 0.412, *d* = 0.03. However, there was a significant group difference in gender composition, χ^2^ = 32.29, df = 3, *p* < 0.001. The proportion of men was significantly greater in the autistic than in the non-autistic groups, χ^2^ = 4.98, df = 1, *p* = 0.026. As the groups were not matched on gender, analyses were conducted to investigate the effects of gender on the variables of interest. Specific data on race/ethnicity and socioeconomic status were not recorded.

As allocation to the autistic and non-autistic groups was based on self-report, all participants were asked to complete the 10-item Autism Spectrum Quotient (AQ-10; Allison et al., 2012) to compare levels of self-reported autistic traits between the two groups. For the AQ-10, scores of 0 denote a lack of or low levels of autistic traits while scores of 10 denote very high levels of autistic traits. Typically, scores of 6 or above pass the threshold for being considered for a formal diagnostic assessment for autism. In the present study, the mean AQ-10 score was significantly higher for the autistic group (M = 7.48, SD = 1.98) than for the non-autistic group (M = 2.72, SD = 1.85), *t*(346) = 22.87, *p* < 0.001, *d* = 2.46. Eighteen participants in the non-autistic group had an AQ-10 score of 6 or more. As removing these participants from the data set did not alter the pattern of results, we opted to retain these participants in the final data set (see Supplementary Material for the same analyses with non-autistic participants with AQ-10 score of over 6 removed).

### Measures

The MBS (Realo et al., 2003) is a short questionnaire which includes questions designed to explore an individual’s perception of their own ability to infer the mental states, emotions, behaviours, characteristics and intentions of others. The 8-item scale includes statements such as ‘Usually, I know beforehand what my conversation partner is going to say’ and ‘I can read people’s intentions in their faces’. Responses are given on a 5-point Likert-type scale ranging from 0 ‘strongly disagree’ to 4 ‘strongly agree’. The scale has good internal consistency with Cronbach’s alphas ranging from 0.70 to 0.82 and a test–retest reliability coefficient of *r* = 0.61 over 3.5 years (Realo et al., 2003).

Four adapted versions of the MBS were created for the current research. The first of these included statements with reference to interactions with non-autistic others only (e.g. ‘I find that it is hard to judge when non-autistic people are lying’), while the second version was identical except that it included statements with reference to interactions with autistic individuals only, for example, ‘I find that it is hard to understand how autistic people feel from their behaviour’. The third version was reworded to ask about participants’ perceptions of how their own minds, emotions and behaviours are understood by others. Accordingly, ‘Usually, I know beforehand what my conversation partner is going to say’ was changed to ‘I think that autistic/non-autistic people can usually tell what I am going to say beforehand in conversation’. Again, using this phrasing, the scale was presented either with specification to respond regarding interactions with non-autistic individuals only, or specification to respond regarding interactions with autistic individuals only. Consequently, the MBS was presented to all participants four times to assess perceptions of their own ability to read autistic others, perceptions of their own ability to read non-autistic others, perceptions of being understood by autistic others and perceptions of being understood by non-autistic others.

To assess prior contact with autistic people, participants were asked if they currently have or have previously had any immediate family members, non-immediate family members, romantic partners or spouses, close friends or acquaintances who identify as autistic. In addition, they were asked if they had worked with autistic individuals. Participants could choose either ‘yes’, ‘no’ or ‘prefer not to say’ for each question.

### Procedure

The survey was created using Qualtrics software (available at http://qualtrics.com) and was distributed using online forums. All participants first completed the questions regarding age, gender and diagnostic status, and answered the questions about their prior contact with autistic people. Following this they completed the AQ-10. Finally, they completed the four versions of the MBS. All participants completed the two questionnaires pertaining to their beliefs about their own mindreading abilities first but the order of these was counterbalanced (i.e. whether they answered with respect to autistic or non-autistic others first). Participants then completed the two versions of the MBS that asked about their perceptions of how others infer the participant’s own thoughts and feelings, with the order of these two versions also being counterbalanced. For all four questionnaires, participants were advised that if they did not have much experience with the target group (autistic/non-autistic people) then they should answer based on their beliefs about what it is like to interact with them. After completing all questions in the survey, participants were thanked for their participation and given further information, useful reading, and contacts.

### Data processing

Prior to processing, the data was checked for any suspicious responses; for example, where the participant selects the same response for every question. Only one such data set was identified and removed prior to analysis. For all four versions of the MBS questionnaire as per previous research, each response, which ranged from ‘highly disagree’ to ‘highly agree’, was coded from 0 to 4. Reverse scoring was used for items that were worded in such a way that a high score (agreement) would express difficulty with mind reading, such as ‘I think that autistic people find it hard to understand how I feel from my behaviour’. This yielded four separate MBS composite scores ranging from 0 to 32 where in all cases higher scores indicated better perceived mindreading ability. Initial checks showed that Cronbach’s alpha for the four scales ranged from α= 0.756, considered to be acceptable (George & Mallery, 2003), to α= 0.838, considered to be good.

For the questions about contact, participants scored 1 for each kind of contact they reported (immediate family members, non-immediate family members, romantic partners or spouses, close friends, acquaintances and people they have worked with). This yielded a total score out of a possible six with higher scores indicating having had more contact.

## Results

**Figure 1.**
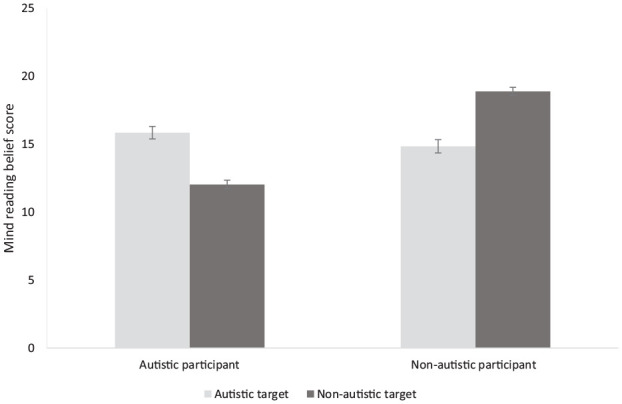
Mean mindreading belief scores for autistic and non-autistic participants when judging their own abilities.

Figure 1 shows the mean mindreading belief score of autistic and non-autistic participants when judging their own ability to read autistic and non-autistic others.

A 2 × 2 mixed analysis of variance (ANOVA) with participant group (autistic or non-autistic) and target group (autistic or non-autistic) as factors revealed a main effect of participant group, *F*(1, 346) = 49.54, *p* < 0.001, η_p_^2^ = 0.13, where non-autistic participants (M = 16.87, SD = 3.35) perceived themselves as better at mindreading others than autistic participants (M = 13.95, SD = 4.39). There was no main effect of the target group but there was a two-way interaction between the participant group and the target group, *F*(1, 346) = 128.56, *p* < 0.001, η_p_^2^ = 0.27. Post-hoc independent samples *t*-tests (with Bonferroni-corrected alpha level of 0.125) revealed that while non-autistic participants reported higher levels of mindreading ability than autistic participants when the target group was non-autistic, *t*(346) = 11.99, *p* < 0.001, *d* = 1.29; autistic participants actually perceived they have higher levels of mindreading ability than non-autistic participants when the target group was designated as autistic, *t*(346) = 1.85, *p* = 0.033, *d* = 0.20, although this was not significant at the corrected alpha level. In line with this, paired samples *t*-tests showed that non-autistic participants believed that they were better at reading non-autistic than autistic targets, *t*(208) = 10.02, *p* < 0.001, *d* = 0.69. Conversely, autistic participants believed that they were better at reading autistic than non-autistic targets, *t*(138) = 6.43, *p* < 0.001, *d* = 0.54.

**Figure 2.**
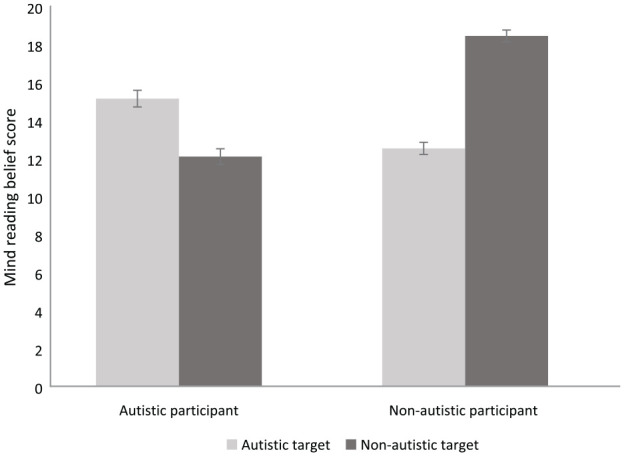
Mean mindreading belief scores for autistic and non-autistic participants when judging other people’s abilities to read them.

Figure 2 depicts the mean mindreading belief scores of autistic and non-autistic participants when judging others’ ability to understand them. A 2 × 2 mixed ANOVA with participant group (autistic or non-autistic) and target group (autistic or non-autistic) as factors revealed a main effect of participant group, *F*(1, 346) = 22.78, *p* < 0.001, η_p_^2^ = 0.06 where non-autistic participants (M = 15.48, SD = 3.34) perceived that they were more readable by others than autistic participants (M = 13.62, SD = 3.87). There was also a main effect of target group, *F*(1, 346) = 18.06, *p* < 0.001, η_p_^2^ = 0.05, whereby overall non-autistic others (M = 15.90, SD = 5.55) were perceived as having better mindreading abilities than autistic others (M = 13.57, SD = 4.97). Finally, there was a two-way interaction between the participant group and the target group, *F*(1, 346) = 176.95, *p* < 0.001, η_p_^2^ = 0.34. Paired samples *t*-tests (Bonferroni-corrected alpha level 0.0125) showed that non-autistic participants believed that non-autistic others were better at reading them than autistic others, *t*(208) = 14.22, *p* < 0.001, *d* = 0.98. Conversely, autistic participants believed that they were better read by autistic than non-autistic others, *t*(138) = 5.65, *p* < 0.001, *d* = 0.48. Similarly, post-hoc independent samples *t*-tests showed that non-autistic participants rated themselves as better read by non-autistic others than autistic participants did, *t*(346) = 12.60, *p* < 0.001, *d* = 1.38, but autistic participants rated themselves as better read by autistic others than non-autistic participants did, *t*(346) = 4.98, *p* < 0.001, *d* = 0.55.

As women were over-represented in the sample, to understand the possible effects of this, we carried out further analysis to explore the impact of gender on responses. The above ANOVAs were repeated with the additional factor of gender. Only men and women were included in this analysis, as the very low number of participants who identified as ‘other’ in the non-autistic group precluded statistical analysis. This yielded a single additional effect in relation to the self-judgements, where gender and participantgroup interacted, *F*(1, 313) = 6.58, *p* = 0.011, η_p_^2^ = 0.02. Independent samples *t*-tests showed that autistic and non-autistic men did not differ in their perceived mindreading abilities. However, autistic women (M = 13.66, SD = 4.39) rated their own mindreading ability as lower than non-autistic women (M = 17.14, SD = 3.15), *t*(237) = 6.23, *p* < 0.001, *d* = 0.97. Notably, gender did not interact with the target group and there was no three-way interaction. For beliefs about other people’s abilities to read oneself, gender had no effects and no interactions.

Contact scores ranged between 0 and 6. Four participants in the non-autistic group and 16 in the autistic group answered that they ‘prefer not to say’ for one or more of the contact questions and were therefore excluded from the analysis as an accurate score for contact could not be calculated. Of the remaining 123 autistic participants, 16 (11.5%) reported having no contact with autistic others (i.e. no immediate family members, non-immediate family members, romantic partners or spouses, close friends, acquaintances and people they have worked with). Twenty-four participants (17.3%) reported one kind of contact, 24 (17.3%) reported two, 26 (18.7%) reported three, 19 (13.7%) reported four, 10 (7.2%) participants reported five and 4 (2.9%) participants reported having all six kinds of contact. Of the remaining 205 non-autistic participants, 60 (28.7%) reported having no contact with autistic others (i.e. no immediate family members, non-immediate family members, romantic partners or spouses, close friends, acquaintances and people they have worked with). Fifty-six participants (26.8%) reported one kind of contact, 44 (21.1%) reported two, 25 (12%) reported three, 16 (7.7%) reported four and 4 (1.9%) participants reported five kinds of contact. No non-autistic participant reported having had all six kinds of possible contact.

To examine whether the amount of self-reported contact with autistic people related to the extent to which the participant believed that they could read autistic people’s minds, for each participant, their mind reading belief score for autistic others was subtracted from their mindreading belief score for non-autistic others. This yielded a difference score that essentially illustrates the participants’ relative beliefs about their ability to read autistic minds compared with non-autistic minds. A positive score would indicate that the participant believes that they are better at reading non-autistic than autistic minds. A negative score would indicate the participant believes that they are better at reading autistic than non-autistic minds. A score of 0 would indicate that the participant believes that they are equally good at reading autistic and non-autistic minds.

Kendall’s tau correlations were used to determine whether the difference scores thus calculated were associated with the amount of contact. For the entire sample, there was a small but significant negative correlation between the amount of contact and difference scores (*b* = –1.84, *p* < 0.001). In other words, those participants who had more contact with autistic others reported slightly less relative difficulty in reading autistic minds in comparison to non-autistic minds. However, when correlations were carried out separately for each group, no significant relationships were found between the amount of contact and the difference in mindreading belief scores for autistic and non-autistic others, for either autistic participants (*b* = –0.06, *p* = 0.544) or non-autistic participants (*b* = –0.06, *p* = 0.205).

## Discussion

This study used modified versions of the MBS (Realo et al., 2003) to explore autistic and non-autistic people’s beliefs about their own ability to read the minds of autistic and non-autistic others, as well as their perceptions of how well autistic and non-autistic others can read their (the participants’) minds. In particular, we aimed to contrast predictions derived from mindreading deficit theories with those derived from the DEP (Milton, 2012). We argued that if participants’ beliefs about their mindreading abilities are in line with autism mindreading deficit theories, then (1) non-autistic people would believe that they are better at reading other minds than autistic people; (2) target group (autistic or non-autistic) would not impact mindreading beliefs. In contrast, predictions derived from the DEP suggest that (3) non-autistic people would believe that they are better at reading non-autistic minds than autistic minds; (4) autistic people would believe that they are better at reading autistic minds than non-autistic minds. For beliefs regarding other people’s mind reading abilities, mindreading deficit theories would lead to the prediction that (5) people would believe that non-autistic people would read them better than autistic people. In contrast, the predictions derived from the DEP suggest that (6) non-autistic people would believe that other non-autistic people would read them better than autistic people; (7) autistic people would believe that other autistic people would read them better than non-autistic people. We also tested the prediction that (8) non-autistic and autistic participants who report having had higher levels of previous contact with autistic people would believe that they could read autistic minds more effectively than those who have had lower levels of previous direct contact.

Non-autistic participants believed themselves to be more adept at mindreading than autistic participants overall in line with prediction 1. However, this effect was driven specifically by the participants’ beliefs about their abilities to read non-autistic others. Non-autistic respondents believed that they were particularly good at reading non-autistic others while autistic respondents believed that they were particularly poor at this. In contrast, when the target group was autistic, autistic participants believed that they were better at mindreading than the non-autistic participants albeit this effect was not significant with a Bonferroni-adjusted alpha level. Thus, prediction 2 was not supported whereas predictions 3 and 4, which hypothesised that each group would believe that they were better at reading their ingroup than their outgroup, were supported.

For beliefs regarding other people’s mindreading abilities, the overall pattern of results was very similar. Overall participants believed that non-autistic others would be better at reading them than autistic others (prediction 5). But, again, this effect was driven by the judgements of the non-autistic participants, who believed that other non-autistic people could read them particularly well. In contrast, autistic participants judged that other autistic people would be able to read their minds better than other non-autistic people. Therefore, the results supported predictions 6 and 7 which captured the idea that both groups would perceive others from their ingroup as better able to read them than others from their outgroup. There was an additional effect in this analysis which we did not predict: there was a main effect of participant group reflecting the fact that autistic participants overall perceived themselves as being harder for others to read. Finally, prediction 8 was not supported, as no relationship was found between the amount of self-reported contact non-autistic participants had had with autistic people and their beliefs about their relative ability to read autistic people.

The findings are consistent with key predictions that can be derived from the DEP (Milton, 2012). First, in line with the results of prior experimental research (Edey et al., 2016; Sheppard et al., 2016), non-autistic participants believed that they have (or would have) more difficulty reading the minds of autistic people than non-autistic others. This suggests that non-autistic people do consider that their mindreading ability is relative to the target in question, and at least to some extent, that non-autistic people are aware that they do not understand autistic people particularly well. Second, not only do autistic people believe that they are better at mindreading when the targets are autistic than when they are non-autistic, but they report abilities at least level with (if not better) than non-autistic people when the target mind in question is autistic. This is consistent with research that shows that autistic people prefer to interact with other autistic people (DeBrabander et al., 2019), and when engaged in interactions with other autistic people they communicate with equal levels of efficacy and rapport as non-autistic people do (Crompton et al., 2020a). The results also correspond with findings from Crompton et al. (2020b) in which autistic adults in interviews reported finding other autistic people easier to read and having a better understanding of other autistic people.

Notwithstanding the interaction between target and perceiver neurotype in determining mindreading beliefs, non-autistic participants did overall self-report greater mindreading ability than non-autistic participants. While in isolation this could be seen as consistent with the supposition that autistic people have poorer mindreading abilities than non-autistic people (Baron-Cohen et al., 1985, 2001), the fact that autistic people did not self-report any deficit in relation to autistic targets is not consistent with a mindreading deficit account. Instead, the main effect of participant group was apparently due to non-autistic participants believing themselves particularly good at reading other non-autistic people and autistic people believing themselves particularly poor at this.

Participants’ answers to the questions regarding other people’s ability to read their (the participant’s) own minds largely followed the same pattern. However, they also yielded an effect of participant group suggesting that overall autistic participants believed that their minds were less readable by others than non-autistic participants. This was surprising insofar as the related effect was not found in the self-ratings, that is there was no overall tendency for participants to self-report having more difficulty reading autistic than non-autistic others. Although direct statistical comparison cannot be made between the two conditions as all participants completed the self-ratings first, the main numerical difference between the self and other ratings appears to be that non-autistic participants rated their own ability to read autistic people as higher than autistic participants rated how well they are read by non-autistic people (although in reality, this pertains to the exact same interaction). Thus, although it can be concluded that non-autistic people are aware that they are not particularly good at reading autistic others, they may nevertheless overestimate their ability in comparison to the perspective of autistic people.

Difficulties reading autistic people were associated with lower contact with autistic people for the whole sample, but not for either group of participants individually. This suggests that the apparent association found for the entire group was due to autistic participants having greater contact with autistic others and reporting less difficulty reading autistic people, without there being a direct relationship between these variables. Therefore, prediction 8 was not supported. The fact that the amount of previous contact with autistic people did not relate to the extent to which participants within a group believed that they have difficulty reading autistic people could be interpreted in various ways. It might be that increased contact between neurotypes does not improve their perceived ability to interpret one another’s behaviour. This is certainly possible, as increased contact with autistic others may in some cases actually increase perceptions of, or awareness of, misunderstandings. Consistent with this, Heasman and Gillespie (2018) found high levels of perceived misunderstanding between autistic people and their close family members.

An alternative possibility is that the measure of contact used in this study was too crude to adequately measure intergroup contact that is relevant to mindreading ability, which is a limitation of this study. We added up the number of different categories of contact that each participant reported, but this does not take into account the frequency of contact, closeness of contact, or number of contacts within a particular category, all of which might impact understanding. Previous research suggests that quality of contact has more effect on attitudes toward autism than just the amount of contact (de Vries et al., 2020; Gardiner & Iarocci, 2014) and the same could be true here. A further related limitation is that we did not collect similar information about participants’ contact with non-autistic people. This could plausibly vary considerably within the sample and could also impact participants’ perceived abilities to read others. Future research using a validated measure of the amount of contact with both autistic and non-autistic people along with measures of contact quality would provide clearer insight into the impact of such contact on the perceived ability to read other minds.

A few other limitations of the research should be highlighted. First, the research was conducted online with participants recruited from a range of websites and forums. While this did result in a large overall sample size, the sample was restricted to those active in the various online communities approached. Moreover, we did not record the nationality or ethnic background of respondents, which would have been valuable in characterising the sample. In both the autistic and non-autistic groups, women made up the bulk of the respondents, which does not represent the true gender ratios within the populations and is often an issue in research as women are more likely to volunteer to participate than men (Smith, 2008). Due to the large sample size, we were able to carry out an exploratory analysis where men versus women gender was included as an additional grouping variable. These yielded a single additional interaction between gender and participant group (autistic or non-autistic) for self-judgements of mindreading ability, which was due to autistic women believing themselves less able to mindread than non-autistic women, but no such difference occurring for the men. As the same pattern did not occur for other-related judgements, this might reflect a gender-specific lack of confidence in one’s own abilities on the part of autistic women as opposed to generalised beliefs about abilities. In line with this, there is some evidence that autistic women may be less self-confident than autistic men in certain other domains including interpersonal and intellectual self-confidence (Sturm & Kasari, 2019). In any case, the lack of further interactions in the analyses implies that the key findings (the interactions between participant and target groups) do apply to both men and women.

Another limitation is that we were not able to independently verify the participants’ self-reported diagnosis, although the AQ-10 scores imply that the autistic group as a whole had higher levels of autistic traits. Concerns over the possibility of fraudulent online research participation have recently been highlighted as a threat to data integrity in autism research (Pellicano et al., 2023). While it is possible that some participants could have lied about their diagnosis, we do not consider this to be particularly likely as there was nothing obvious to gain by lying: participation was voluntary with no financial compensation, and participants could answer the survey regardless of whether they were formally diagnosed, identified as autistic with no diagnosis, or neither. Moreover, as the pattern of results differed between the two groups largely in line with our predictions, this does suggest that the groups that were recruited were genuinely different from one another.

Finally, it is important to acknowledge that this study focused on participants’ *beliefs* about the mindreading abilities of themselves and others, and we did not include any direct measure of mindreading ability. Therefore, we cannot know to what extent participants’ self-reports correspond to the ‘truth’ about their own mindreading skills. Indeed, previous research with non-autistic participants has found no relationship between self-reported mindreading ability on the MBS and performance on experimental measures of mindreading including recognition of facial and verbal emotional expressions and speech, and recognition of the personality traits of a stranger (Realo et al., 2003). This could be evidence that (non-autistic) people are unaware of their own ‘true’ mindreading ability, perhaps reflecting a more generalised tendency that they have to overestimate their own competence across a wide variety of performance domains (Dunning et al., 2003, for a review). However, as argued earlier, this rests on the supposition that performance on mindreading ‘tasks’ adequately indexes everyday mindreading ability. In relation to autism, performance on sociocognitive tasks has little impact on real-world social skills in this group (Morrison et al., 2020; Sasson et al., 2020), in line with the possibility that they do not fully capture real-world mindreading skills. It would be interesting to see whether participants’ self-reported mindreading beliefs do a better job of predicting social outcomes.

Regardless of whether participants’ mindreading beliefs reflect ‘reality’, beliefs are powerful insofar as they impact behaviour. If both groups of participants believe that they do not understand, and are not understood by, members of the other group then this could have a significant impact on relations between the groups. It has been suggested that readability may be linked to social favourability in autism (Alkhaldi et al., 2019), and this may be because people feel less liking for individuals whom they think they cannot read (Anders et al., 2016). This in turn could have further adverse consequences for autistic people, such as being socially isolated (at least from the non-autistic majority) and associated issues with mental health (Mitchell et al., 2021).

## Conclusion

Based on a relatively large sample, the beliefs of autistic and non-autistic people about mindreading abilities themselves and others fall largely in line with predictions that can be derived from the DEP (Milton, 2012) as opposed to mindreading deficit theories. While this is in accordance with previous qualitative studies (Crompton et al., 2020b), the much larger sample size here suggests that these beliefs may be widespread. This also corroborates previous research that has found that specifying the reference group (as autistic or non-autistic) systematically impacts autistic participants’ answers on questionnaires designed to measure autistic traits (Gernsbacher et al., 2017). The findings also yield new knowledge that it is not just autistic people who believe that mindreading difficulties are a two-way street: the responses of the non-autistic group were in line with predictions derived from the DEP too.

## Supplemental Material

sj-docx-1-aut-10.1177_13623613231211457 – Supplemental material for Mindreading beliefs in same- and cross-neurotype interactions Supplemental material, sj-docx-1-aut-10.1177_13623613231211457 for Mindreading beliefs in same- and cross-neurotype interactions by Elizabeth Sheppard, Sophie Webb and Helen Wilkinson in Autism

## Notes

1. Although ‘mindreading’ as a term could be problematic in invoking connotations of telepathy, its meaning is well understood within psychology, and, unlike ‘Theory of Mind’ does not imply any particular kind of mental process. In this article, we opted to use this term, in line with the name of the measurement scale that was used.
