# Supplementary material for: Mindreading beliefs in same- and cross-neurotype interactions
Source: Autism. 2023 Nov 18;28(7):1828–37. doi: 10.1177/13623613231211457 (PMC11191369; doi:10.1177/13623613231211457)
Supplement: sj-docx-1-aut-10.1177_13623613231211457 – Supplemental material for Mindreading beliefs in same- and cross-neurotype interactions [file sj-docx-1-aut-10.1177_13623613231211457.docx]

**Supplemental material for Mindreading beliefs in same- and cross-neurotype interactions**

Analyses with non-autistic participants with AQ-10 score of 6 or above removed:

A 2 x 2 mixed ANOVA with participant group (autistic or non-autistic) and target group (autistic or non-autistic) as factors revealed a main effect of participant group, *F*(1,328) = 62.59, *p* < .001, *η_p_^2^* = 0.16, where non-autistic participants (*M* = 17.15, *SD* = 2.96) perceived themselves as better at mindreading others than autistic participants (*M* = 13.95, *SD* = 4.39). There was no main effect of target group but there was a two-way interaction between participant group and target group, *F*(1,328) = 127.31, *p* < .001, *η_p_^2^* = 0.28. Post-hoc independent samples t-tests (with Bonferroni corrected alpha level of 0.125) revealed that while non-autistic participants reported higher levels of mindreading ability than autistic participants when the target group was non-autistic, *t*(328) = 12.73, *p* < .001, *d* = 1.50; there was no difference between the groups when the target group was designated as autistic. In line with this, paired samples t-tests showed that non-autistic participants believed that they were better at reading non-autistic than autistic targets, *t*(190) = 9.91, *p* < .001, *d* = 0.72. Conversely autistic participants believed that they were better at reading autistic than non-autistic targets, *t*(138) = 6.41, *p* < .001, *d* = 0.54.

A 2 x 2 mixed ANOVA with participant group (autistic or non-autistic) and target group (autistic or non-autistic) as factors revealed a main effect of participant group, *F*(1,328) = 27.70, *p* < .001, *η_p_^2^* = 0.08 where non-autistic participants (*M* = 15.66, *SD* = 3.16) perceived that they were more readable by others than autistic participants (*M* = 13.62, *SD* = 3.87). There was also a main effect of target group, *F*(1,328) = 21.77, *p* < .001, *η_p_^2^* = 0.06 whereby overall non-autistic others (*M* = 15.96, *SD* = 5.53) were perceived as having better mindreading abilities than autistic others (*M* = 13.63, *SD* = 4.98). Finally there was a two-way interaction between participant group and target group, *F*(1,328) = 184.64, *p* < .001, *η_p_^2^* = 0.36. Paired samples t-tests (Bonferroni-corrected alpha level 0.0125) showed that non-autistic participants believed that non-autistic others were better at reading them than autistic others, *t*(190) = 14.49, *p* < .001, *d* = 1.05. Conversely autistic participants believed that they were better read by autistic than non-autistic others, *t*(138) = 5.65, *p* < .001, *d* = 0.48. Similarly post-hoc independent samples t-tests showed that non-autistic participants rated themselves as better read by non-autistic others than autistic participants did, *t*(328) = 13.50, *p* < .001, *d* = 1.51; but autistic participants rated themselves as better read by autistic others than non-autistic participants did, *t*(328) = 4.85, *p* < .001, *d* = 0.54.

As women were over-represented in the sample, to understand possible effects of this, we carried out further analysis to explore the impact of gender on responses. The above ANOVAs were repeated with the additional factor of gender. Only men and women were included in this analysis, as the very low number of participants who identified as “other” in the non-autistic group precluded statistical analysis. This yielded a single additional effect in relation to the self-judgments, where gender and participant group interacted, *F*(1,297) = 4.77, *p* = .03, *η_p_^2^* = 0.02. Independent samples t-tests showed that autistic and non-autistic men did not differ in their perceived mindreading abilities. However, autistic women (*M* = 13.66, *SD* = 4.39) rated their own mindreading ability as lower than non-autistic women (*M* = 17.30, *SD* = 2.93), *t*(227) = 6.58, *p* < .001, *d* = 1.05. Notably, gender did not interact with target group and there was no three-way interaction. For beliefs about other people’s abilities to read oneself, gender had no effects and no interactions.

Contact scores ranged between 0 and 6. Three participants in the non-autistic group and 16 in the autistic group answered that they “prefer not to say” for one or more of the contact questions and were therefore excluded from the analysis as an accurate score for contact could not be calculated. Of the remaining 123 autistic participants, 16 (11.5%) reported having no contact with autistic others (i.e. no immediate family members, non-immediate family members, romantic partners or spouses, close friends, acquaintances, people they have worked with). Twenty-four participants (17.3%) reported one kind of contact, 24 (17.3%) reported two, 26 (18.7%) reported three, 19 (13.7%) reported four, 10 (7.2%) participants reported five, and 4 (2.9%) participants reported having all six kinds of contact. Of the remaining 188 non-autistic participants, 49 (26.1%) reported having no contact with autistic others (i.e. no immediate family members, non-immediate family members, romantic partners or spouses, close friends, acquaintances, people they have worked with). Fifty-three participants (28.2%) reported one kind of contact, 42 (22.3%) reported two, 24 (12.8%) reported three, 16 (8.5%) reported four, and four (2.1%) participants reported five kinds of contact. No non-autistic participant reported having had all six kinds of possible contact.

To examine whether amount of self-reported contact with autistic people related to the extent to which the participant believed that they could read autistic people’s minds, for each participant, their mind reading belief score for autistic others was subtracted from their mindreading belief score for non-autistic others. This yielded a difference score that essentially illustrates the participants’ relative beliefs about their ability to read autistic minds compared with non-autistic minds. A positive score would indicate that the participant believes that they are better at reading non-autistic than autistic minds. A negative score would indicate the participant believes that they are better at reading autistic than non-autistic minds. A score of 0 would indicate that the participant believes that they are equally good at reading autistic and non-autistic minds.

Kendall’s tau correlations were used to determine whether the difference scores thus calculated were associated with amount of contact. For the entire sample, there was a small but significant negative correlation between amount of contact and difference scores ($\tau$*b* = -1.74, *p* < .001). In other words, those participants who had more contact with autistic others reported slightly less relative difficulty in reading autistic minds in comparison to non-autistic minds. However, when correlations were carried out separately for each group no significant relationships were found between amount of contact and the difference in mindreading belief scores for autistic and non-autistic others, for either autistic participants ($\tau$*b* = -0.05, *p* = .333), or non-autistic participants ($\tau$*b* = -0.10, *p* = .124).
